# Bisamide Derivative of Dicarboxylic Acid Contributes to Restoration of Testicular Tissue Function and Influences Spermatogonial Stem Cells in Metabolic Disorders

**DOI:** 10.3389/fcell.2020.562358

**Published:** 2020-12-03

**Authors:** Angelina Pakhomova, Olga Pershina, Vladimir Nebolsin, Natalia Ermakova, Vyacheslav Krupin, Lubov Sandrikina, Edgar Pan, Darius Widera, Alexander Dygai, Evgenii Skurikhin

**Affiliations:** ^1^Laboratory of Regenerative Pharmacology, Goldberg ED Research Institute of Pharmacology and Regenerative Medicine, Tomsk National Research Medical Centre of the Russian Academy of Sciences, Tomsk, Russia; ^2^“PHARMENTERPRISES Ltd.,” Moscow, Russia; ^3^Stem Cell Biology and Regenerative Medicine Group, School of Pharmacy, University of Reading, Reading, United Kingdom

**Keywords:** metabolic disorders, hypogonadism, spermatogenesis, infertility, bisamide derivative of dicarboxylic acid, spermatogonial stem cells, regeneration

## Abstract

Metabolic syndrome can lead to several challenging complications including degeneration of the pancreas and hypogonadism. Recently, we have shown that Bisamide Derivative of Dicarboxylic Acid (BDDA) can contribute to pancreatic restoration in mice with metabolic disorders via its positive effects on lipid and glucose metabolism, and by increasing the numbers of pancreatic stem cells. In the present study, we hypothesized that BDDA might also be effective in restoring hypogonadism caused by metabolic syndrome. Experiments were performed on male C57BL/6 mice with hypogonadism, where metabolic disorders have been introduced by a combination of streptozotocin treatment and high fat diet. Using a combination of histological and biochemical methods along with a flow cytometric analysis of stem and progenitor cell markers, we evaluated the biological effects of BDDA on testicular tissue, germ cells, spermatogonial stem cells *in vitro* and *in vivo*, as well as on fertility. We demonstrate that in mice with metabolic disorders, BDDA has positive effects on spermatogenesis and restores fertility. We also show that BDDA exerts its therapeutic effects by reducing inflammation and by modulating spermatogonial stem cells. Thus, our results suggest that BDDA could represent a promising lead compound for the development of novel therapeutics able to stimulate regeneration of the testicular tissue and to restore fertility in hypogonadism resulting from complications of metabolic syndrome.

## Introduction

The International Association of Diabetes defines metabolic syndrome (MS) as a combination of abdominal obesity, insulin resistance, hyperglycemia, dyslipidemia, arterial hypertension, violation of the hemostatic system, and chronic subclinical inflammation (Standl, 2005; International Diabetes Federation, 2016). MS is a complex set of symptoms which is endemic in industrialized countries and can be found in every fifth adult person. Its prevalence is expected to increase by 50% in the next 25 years (Ford et al., 2002; Vancampfort et al., 2013). Notably, there is also a considerable and steady increase in the prevalence of MS among adolescents and young people (Rask-Madsen and Kahn, 2012). In addition to prolonged and excessive fat intake, genetic predisposition, and hypodynamia represent the main etiological factors of MS (Matsushita and Dzau, 2017). The socioeconomic impact of MS is mainly associated with its complications, including but not limited to hypogonadism in men, cardiovascular disease, and type 2 diabetes (World Health Organization [WHO], 2014, 2016). In men with MS, a low level of testosterone often correlates with disturbances of carbohydrate and fat metabolism (Dhindsa, 2004; Kapoor et al., 2007; Wespes, 2013; Lamm et al., 2016; Dimopoulou et al., 2018). Metabolic disorders (MD) in MS can also lead to the development of hypogonadism with a prevalence reaching 50–75% (Lamm et al., 2016). Since hypogonadism can exacerbate metabolic disorders (Kupelian et al., 2006; Selvin et al., 2007; Ebrahimi and Christ-Crain, 2016; Lamm et al., 2016), the relationship between metabolic disorders and hypogonadism is bi-directional (Dhindsa, 2004; Kupelian et al., 2006). Laaksonen et al. (2004) showed that low levels of free testosterone increased the risk of MS 2–3-fold compared to men with normal testosterone levels. Since the central element of hypogonadism is the reduction of testosterone concentration, its treatment is based on filling the deficit of this hormone (Kupelian et al., 2006). Due to the substitutive nature of this treatment, it is carried out continuously. Notably, current treatment of hypogonadism can only reduce the symptoms of androgen deficiency but is not able to cure it (Lunenfeld et al., 2015).

A possible alternative to hormonal therapy in male patients with MS and hypogonadism could be a drug which selectively targets testicular stem and progenitor cells. Novel high-effective metal chelators are currently being explored as therapeutic agents in regeneration of organs affected by diabetic degeneration (Cooper et al., 2004; Cooper, 2011, 2012; Lu et al., 2013). Derivatives of bisamides of dicarboxylic acids including bisamide derivative of dicarboxylic acid (BDDA) can chelate metal ions including zinc, copper, iron, magnesium, and calcium (Nebolsin et al., 2016, 2018). Based on our previous studies showing that BDDA has regenerative properties and restores the function of multiple tissues and organs (Nebolsin et al., 2016, 2018; Pakhomova et al., 2020), we aimed to assess the efficacy of BDDA on hypogonadism resulting from MD in male C57BL/6 mice. The effects of BDDA on testicular stem and progenitor cells were evaluated *in vivo* and *in vitro.*

## Materials and Methods

### Animals

The experiments were performed on male and female C57BL/6 mice obtained from the nursery of the Surgical Bio-modeling Department of the Goldberg ED Research Institute of Pharmacology and Regenerative Medicine (veterinary certificate available) housed under pathogen-free conditions with food and water *ad libitum*. All animal experiments were carried out in accordance with the European Convention on the protection of vertebrates used in experiments or for other scientific purposes. The study was approved by the laboratory animal control Committee of the Goldberg ED Research Institute of Pharmacology and Regenerative Medicine, Tomsk NRMC (IACUC Protocol No. 114062016).

### Modeling of Metabolic Disorders

**FIGURE 1:**
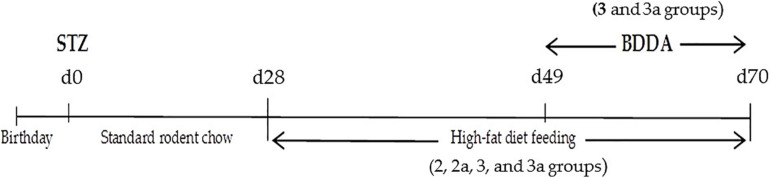
Graphical scheme of the protocol for inducing MD.

Experimental MD were modeled by a single subcutaneous injection of streptozotocin into the withers (Sigma, St. Louis, MO, United States) at a dose of 200 mg/kg in 30 μl of phosphate buffer 1 day after birth. A special diet enriched with heavy saturated fats (Ssniff EF R/M with 30% Fat, Soest, Germany, ref. No. E15116-34) (Fujii et al., 2013; Kaya-Dagistanli and Ozturk, 2013; Patil et al., 2014; Pakhomova et al., 2020) was administered on postnatal d28–d70. Introduction of streptozotocin was set as d0 of the experiment (Figure 1). Age matched animals that received streptozotocin were from the multiple litters. Mice were co-housed (5–6 mice per cage) and entrained to a reverse 12 h light/12 h dark cycle. During the 28 days, all mice had *ad libitum* access to an unpurified standard rodent chow (Ssniff Control diet, Soest, Germany, kat. No̱. E15000-04Çà). The 20 mice were maintained on the standard chow diet for the entire study and served as a healthy control group. The remaining mice were given a chow standard diet *ad libitum* within 28 days then they had been fed a high-fat diet for 6 weeks to induce metabolic disorders (Figure 1). Mice were randomized according to bodyweight on d49. The time points for the experiments were selected as described previously (Fujii et al., 2013; Pakhomova et al., 2020).

### BDDA

**FIGURE 2:**
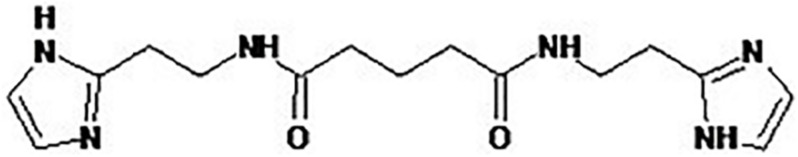
Structural formula of Bisamide Derivative of Dicarboxylic Acid (BDDA).

BDDA or Treamid (XC268BG), chemical formula N1,N5-bis [2-(1H-imidazole-2-Il)ethyl]glutaramide (Figure 2), was kindly provided by PHARMENTERPRISES Ltd. (Nebolsin et al., 2016, 2018; Pakhomova et al., 2020). BDDA was dissolved in 0.5% carboxymethylcellulose solution (vehicle). BDDA was administered daily intragastrically at a dose of 10 mg/kg on d49–d70 (Figure 1). The BDDA dose was selected according to a previous report (Nebolsin et al., 2016, 2018; Pakhomova et al., 2020). Control animals received the solvent at an equivalent volume. We made adjustments weekly to the amount of BDDA given throughout the course of the experiment due to fluctuations in body weight. Control animals received the solvent at an equivalent volume.

### Experimental Groups

Mice without MD receiving saline solution formed the control group (group 1 and 1a–control) (Table 1). Mice with metabolic disorders were allocated to two experimental groups: mice with metabolic disorders (group 2 and 2a–metabolic disorder) and mice with metabolic disorders treated with BDDA (group 3 and 3a–metabolic disorders treated with BDDA). All the mice from groups 1, 2, and 3 were sacrificed on d70 with CO_2_. The remaining mice from groups 1a, 2a, and 3a were sacrificed on d80 with CO_2_.

**TABLE 1:** Groups of animals in the experiments *in vivo.*

	Intact control	Metabolic disorders	Mice with metabolic disorders treated with BDDA
Males	Group 1 (*n* = 10)	Group 2 (*n* = 10)	Group 3 (*n* = 10)
Males	Group 1a (*n* = 10)	Group 2a (*n* = 10)	Group 3a (*n* = 10)

### Parameters Connected With Glucose Homeostasis in the Serum

Glucose level in the serum was evaluated on d28, d35, d42, d49, d56, and d70 using Accu-Chek Performa Nano glucometer (Roche Diagnostics GmbH, Mannheim, Germany). Insulin levels in the serum were measured on d70 using an ELISA kit according to manufacturer’s instructions (Cusabio Biotech Co., Ltd., China).

### Morphological Assessment of the Testes

Morphological assessment of the testes was carried out on d70. For this purpose, the tissue was fixed in a 10% solution of neutral formalin, processed through ascending concentrations alcohols to xylene and poured into paraffin. Dewaxed, 5 μm thick sections were stained with hematoxylin and eosin (Cardiff et al., 2014; Skurikhin et al., 2017). Micro-preparations from each animal were examined under a light microscope Axio Lab.A1 (Carl Zeiss, MicroImaging GmbH; Göttingen, Germany) at 100× and 400× magnifications.

Hematoxylin-eosin was performed to assess testes architecture and potential presence of inflammatory cells (neutrophils, lymphocytes, and macrophages). The state of tubular apparatus, morphological features of Leydig cells and the state of stroma were evaluated on the testicular preparations. In addition, the presence of inflammatory infiltrate and the state of the microvasculature were assessed on preparations of the testes.

### Sperm Counts

Sperm counts were performed as described previously (Welborn et al., 2015; Shi et al., 2017). Briefly, the caudal region of the epididymis was dissected, and placed in 500 μl of phosphate-buffered saline (PBS) containing 1% bovine serum albumin (Sigma, St. Louis, MO, United States) heated to 37°C. The caudal epididymis was splayed open using a back-cutting method and then left undisturbed for 30 s (giving sperm time to escape into liquid). Subsequently, 5 μl of sperm-containing liquid was diluted in 45 μl of PBS containing 1% bovine serum albumin (1:10 dilution), and 10 μl of this dilution was placed in the center of a Gorjaev’s count chamber. Motile and non-motile cells were counted to determine sperm motility and differences in total sperm populations.

Total number of sperms and the number of mobile sperms per epididymis were determined on d70 using an Axio Lab microscope.A1 (Carl Zeiss, MicroImaging GmbH; Göttingen, Germany) at a magnification of 400×. A minimum of 200 spermatozoa was assessed.

### Fertility Study

Healthy female mice were placed to intact control male mice (group 1a), untreated (group 2a) and treated BDDA (group 3a) males with metabolic disorders for 10 days (from d70 to d80) (Table 1). In an individual cage, the male/female ratio was 1:2. In each group, there were 20 females for 10 males. During the study period, BDDA at a dose of 10 mg/kg was further administered intragastically once a day to mice males of metabolic disorders + BDDA group (group 3a). On d80 females and males were placed in different cages. Females were sacrificed in a CO_2_ chamber on the 18th day of pregnancy. The index of fecundity (IF) was calculated for the assessment of fecundity (Muroi et al., 2017):

$$\begin{array}{c} \mathrm{IF}=\mathrm{number}\mathrm{of}\mathrm{fertilized}\mathrm{females}/\\ \;\mathrm{total}\mathrm{number}\mathrm{of}\mathrm{females}\mathrm{in}\mathrm{the}\mathrm{group}\times 100\% \end{array}$$

### Flow Cytometric Analysis

Mononuclear cells from testes were obtained as previously described on d70 (Skurikhin et al., 2017). Receptors expression on surface membranes of murine mononuclear cells derived from testes was analyzed using BD surface markers (BD Biosciences, United States). Fc-receptors were first blocked by pre-incubation of cells for 10 min with unconjugated anti-CD16/CD32 antibodies (eBioscience, San Diego, CA, 1:50 dilution) in 50 μL of 0.1% saponin (Sigma-Aldrich, St. Louis, MO) and 1% bovine serum albumin (BSA) (Sigma-Aldrich, St. Louis, MO) in PBS per tube. After the pre-incubation, cells suspensions were stained with fluorophore-conjugated monoclonal anti-Mouse antibodies: CD9APC (1:50), CD24APC (1:50), CD29FITC (1:50), CD31APC (1:100), CD45APC-Cy-7 (1:100), CD51PE (1:50), CD52FITC (1:50), CD90Per-Cy5.5 (1:100), CD117 (c-kit)PE-Cy7 (1:100), CD309 (Flk-1)APC (1:50), CD326PE (1:50), and Sca1Per-Cy5.5 (1:50) (all Becton Dickinson, San Jose, CA, United States). The antibody CD49f FITC (1:50, Becton Dickinson, San Jose, CA, United States) was rat Anti-Human with reactivity for mouse. All antibodies were titrated to determine their optimal staining concentration. Appropriate isotype controls were used. We incubated tubes in the dark for 30 min. Labeled cells were washed twice with 500 μL of FACSFlow (Becton Dickinson, San Jose, CA, United States). Compensation adjustments were performed with single color positive controls. All samples were run on a Becton Dickenson FACSCanto II flow cytometer. The instrument was set up and standardized using BD Cytometer Setup and Tracking (CS&T) procedures according to manufacturer specifications. 100,000 events per tube were collected and saved as list mode files. Data were analyzed using *FACSDiva 8.0* software.

### Dissociation of Testicular Tissue and Isolation of Stem and Progenitor Cells for Studies *in vitro*

On d70, the testicles of donor mice were isolated using sterile instruments in sterile conditions in the cold without liquid, then the protein shell of the testicles was removed, and the isolated testicular tissue was crushed. Tubule fragments were washed using chilled PBS containing 1% bovine serum albumin (Sigma, St. Louis, MO, United States) for 10 min at 60 g. The precipitate in DMEM (Sigma, St. Louis, MO, United States) containing type IV collagenase (Sigma, St. Louis, MO, United States) at a concentration of 1 mg/ml was resuspended and incubated at 37°C for 5 min. After incubation, the cells were washed twice in PBS without calcium and magnesium at 200 g for 3 min and resuspended in trypsin-EDTA solution (0.25% trypsin, 1 μmol EDTA). 2 ml of DNase (80 μ/ml) were added to the obtained cellular suspension. One minute later the reaction was stopped by adding fetal bovine serum (FBS), after which the cell suspension was filtered using a nylon filter with a pore size of 40 μm, centrifuged at 600 g for 5 min. Using flow cytometry in the obtained suspension of testicular cells, the number of spermatogonal stem cells (CD117^–^CD29^+^CD90^+^; CD117^+^CD29^+^CD90^+^; CD51^–^CD24^+^CD52^+^), and epithelial precursors (CD45^–^CD31^–^Sca1^+^CD49f^+^) was determined. The cellular suspension was divided into two parts. The first part of cellular suspension was used in *in vitro* experiments, and the second one in transplantation experiments.

### Culture of Stem and Progenitor Cells Isolated From the Testes

After the isolation of stem and progenitor cells from the testes, self-maintenance potential has been evaluated *in vitro*. To increase the efficiency of culture of spermatogonial stem cells, endothelial or epithelial precursors, the precursors of stromal cells, a monolayer of adherent bone marrow cells from C57BL/6 mice was previously prepared in conditions of optimal functioning (*n* = 6) using a standard Protocol. The cells have been cultivated for 7–10 days at 37°C, 100% humidity and 5% CO_2_ as described before (Benavides-Garcia et al., 2015; Muroi et al., 2017), after which myelokaryocytes were removed from the plastic using Trypsin-EDTA solution (0.05% trypsin, 0.53 μmol EDTA (Sigma, St. Louis, MO, United States) for 10 min at 37°C. The collected cells were centrifuged at 300 g for 5 min and pellet was resuspended in growth medium. Myelocariocytes were transferred to culture plates coated with gelatine at a cell density of 250 cells × mm^–2^, and the feeder cells were incubated for 24 h at 37°C, 100% humidity and 5% CO_2_. In the following, the culture medium was removed and a fresh solution of doxorubicin (Pharmachemie B. V., Haarlem, Netherlands) at a concentration of 10 μg × ml^–1^ was added to inactivate cell growth of the adherent myelocariocytes, after which the cells were incubated for 4 h at 37°C, 100% humidity and 5% CO_2_. Subsequently, the medium was removed, and the wells were washed with DMEM (Sigma, St. Louis, MO, United States). The growth medium (prepared as described in Kanatsu-Shinohara et al., 2016), SSCs, endothelial, and epithelial precursors of the testicles isolated as described above, were added to the culture plates. The growth medium consisted of 50% of Dulbecco’s Modified Eagle’s Medium (Sigma, St. Louis, MO, United States), 50% of Dulbecco’s Modified Eagle’s Medium: F-12 (Sigma, St. Louis, MO, United States), 2 ìÌ L-glutamine Sigma, St. Louis, MO, United States), 0.5% BSA (Sigma, St. Louis, MO, United States), antibiotic solution (penicillin/streptomycin 100 U × ml^–1^ and 100 μg × ml^–1^; Sigma, St. Louis, MO, United States), D glucose 6 mg × ml^–1^ (Sigma, St. Louis, MO, United States), β-estradiol 30 ng × ml^–1^ (Sigma, St. Louis, MO, United States), progesterone 60 ng × ml^–1^ (Sigma, St. Louis, MO, United States), 1% ETS (Sigma, St. Louis, MO, United States), bovine holo-transferrin 100 μg × ml^–1^ (Sigma, St. Louis, MO, United States), insulin 25 μl × ml^–1^ (Sigma, St. Louis, MO, United States), ascorbic acid 100 ìÌ (Sigma, St. Louis, MO, United States), 30 μg × ml^–1^ sodium pyruvate (Sigma, St. Louis, MO, United States), 5 × 10^–5^ M2-mercaptoethanol (Thermo Fisher Scientific, Waltham, Massachusetts, United States), 10 ng × ml^–1^ human basic fibroblast growth factor (Sigma, St. Louis, MO, United States), 10 ng × ml^–1^ recombinant rat glial cell line-derived neurotrophic factor (GDNF) (R&D Systems, Minneapolis, MN), and 20 ng × ml^–1^ epidermal growth factor (Sigma, St. Louis, MO, United States). The final cell concentration for the cultivation was 0.5 × 10^6^/ml and the cells were cultivated for 7 days at 37°C, 100% humidity and 5% CO_2_. At the end of the cultivation, colonies were collected, washed twice with DMEM and processed for flow cytometry to assess the expression of CD9, CD24, CD31, CD45, CD49f, CD51, CD52, CD90, CD117, and Sca1. Based on the results of flow cytometry, conclusions were made about a potential for self-renewal.

### Statistical Analysis

Statistical analysis was performed using SPSS statistical software (version 15.0, SPSS Inc., Chicago, IL, United States). Data were analyzed and presented as means ± standard error of mean. A two-sided unpaired Student *t*-test (for parametric data) or Mann-Whitney test (for non-parametric data) was used according to distribution. A *P*-value of less than 0.05 (by two-tailed testing) was considered an indicator of statistical significance.

## Results

### Effects of BDDA on Biochemical Parameters

We have previously shown that streptozotocin and a high fat diet causes an increase in the concentrations of glucose, insulin, triglycerides (TG) and very-low-density lipoproteins (VLDL) in the serum of male C57BL/6 mice, as well as increases the body mass index (BMI) (Pakhomova et al., 2020). Overall, these changes correspond to the clinical patterns of MD. BDDA introduction reduced the BMI, glucose level, insulin level, the atherogenic index, and the concentration of TG, low-density lipoproteins LDL, and VLDL in serum (Pakhomova et al., 2020). The streptozotocin introduction and high fat diet increased food consumption (Supplementary Table S1). We have further demonstrated that BDDA improves the physiological function of the pancreas in animals with MD.

In the present study, we demonstrate that mice who received streptozotocin and a high fat diet have an increased level of glucose (3.8 times), and insulin (81 times) (Supplementary Figure S1).

### Effects of BDDA on Tissue Morphology

**FIGURE 3:**
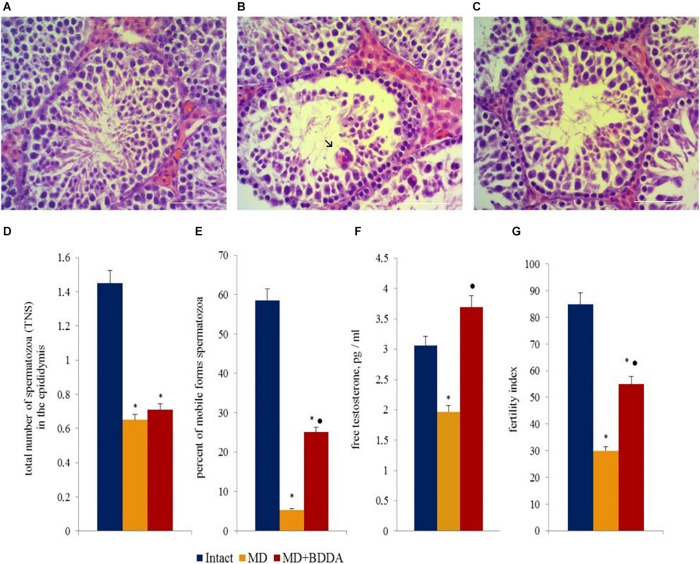
Photomicrographs of representative testis sections **(A–C)** obtained from male C57BL/6 mice on d70. The arrows indicate “seed balls.” Tissue was stained with hematoxylin-eosin. **(A)** Section from control group; **(B)** Section from mice with MD; **(C)** Section from mice with MD treated with BDDA between d49 and d70. At least 10 photomicrographs of the testicular tissue at × 400 magnification were taken for each experimental animal from all experimental groups (group 1, 2, and 3). Scale bar 50 μm. **(D,E)** Total number of spermatozoa and the percentage of mobile forms of spermatozoa in the epididymis of male C57BL/6 mice with MD on d70. **(F)** Free testosterone level in blood serum on d70. **(G)** Fertility index of mice with MD on d80. Groups: intact—control group from intact mice, MD—mice with MD, MD + BDDA—mice with MD treated with BDDA. Results are presented as the mean ± SEM. *Significance of difference compared with control (*p* < 0.05); •significance of difference compared with the MD group (*p* < 0.05).

Analysis of testes sections (Figure 3 and Supplementary Figure S2) revealed that moderate destructive changes in the tubular apparatus of the testes in mice of group 2 occurred on d70, including a decrease in the number of layers of spermatogenic epithelium in the seminiferous tubules, edema of interstitial tissue, and hyperemia of vessels. We have also detected degenerative changes including cytoplasm vacuolization, and hyperchromia of the nuclei of Leydig cells (Figures 3A,B). In several convoluted tubules, empty layers of spermatogenic epithelium or disappearance of mature forms of germ cells were observed. We also observed a presence of single tubules with cell debris in the lumen, consisting of dead sperm and spermatids. Moreover, “seed balls”—large structures with multiple, often pyknotic nuclei or their fragments with intensely colored cytoplasm were detected in a small amount within the desquamated epithelium. BDDA increased the number of layers of spermatogenic epithelium in the seminiferous tubules in mice with MD on d70, while germ cells were found in the convoluted tubules (Figure 3C and Supplementary Figure S3).

### Effects of BDDA on Testosterone Levels in Blood Serum

Testosterone is the key hormone of gonad development and spermatogenesis (Zheng et al., 2014). The hormone is produced by Leydig cells and in small amounts by the adrenal cortex (Zang et al., 2017). Modeling of MD reduced the levels of testosterone in the serum in group 2 (by 44.5%) relative to the control group on d70 (Figure 3F). BDDA increased serum testosterone levels group 3. The values of the index were higher than those in group 2 (87%) and group 1 (20.9%). However, the BDDA did not increase the testosterone level in the normal male mice (Supplementary Figure S4).

### The Effect of BDDA on Sperm Cells

Previously, we have demonstrated that MD results in the development of pathological morphological alterations characteristic for hypogonadism in testicular tissue of male C57BL/6 mice (Skurikhin et al., 2017). Simultaneously, infertility progressed in parallel to a drop in the total number of testicular germ cells. Under these conditions, spermatogenesis was maintained by SSCs. MD caused a significant decrease in the number of mobile sperm cells and the total number of sperm in the epididymis on d70 (Figures 3D,E). Simultaneously with asteno—and oligozoospermia in group 2, the fertility index decreased compared to group 1 (64.7%). In mice with MD, BDDA increased the number of mobile sperms (4.7 times) in the epididymis and fertility index (83%) compared to group 2 (Figures 3D,E,G).

### Impact of BDDA on the Cytokine Profile

In order to assess the potential impact of MD and BDDA on the cytokine levels in the testes, an ELISA assay has been performed. In animals with MD introduced by a single streptozotocin application and long-term consumption of fat, a significant increase of interleukins 2 (IL-2, 4.7 times), IL-4 (4 times), IL-5 (2.9 times), IL-10 (2.3 times), IL-17 (3.9 times), and IL-23 (1.7 times), as well as IL-1ra (3.9 times) and as TGF-1β (2.4 times) was detected in the homogenates of the testes of group 2. Interestingly, levels of IL-6 (3 times) and TNF-α (1.4 times) decreased in this group (Table 2). In our previous study, we have shown that BDDA introduction increases serum levels of IL-1ra, IL-4, IL-5, IL-13, and IL-23 in mice with MD compared to animals without MD (Supplementary Figure S5; Pakhomova et al., 2020). In contrast, the levels of IL-1β, IL-1ra, IL-5, IL-23, and TNF-α were decreased (Supplementary Figure S5; Pakhomova et al., 2020). In testes, BDDA increased the levels of IL-1β (1.8 times), IL-4 (2.7 times), IL-10 (1.4 times), and IL-23 (1.4 times) in mice of group 3, but decreased the concentration of IL-5 (2.4 times) and IL-17 (2.5 times).

**TABLE 2:** Levels of ILs, TNF-alpha, TGF-1beta (pg/ml), and IL-1ra (pg/ml) in the testicular tissue homogenates of male C57BL/6 mice on d70 (M ± m, *n* = 10).

Index	Intact control	Metabolic disorders	Metabolic disorders + BDDA
IL-1beta	453.84 ± 12.65	479.92 ± 15.02	883.94 ± 13.85^∗^•
IL-1ra	1188.12 ± 17.09	4676.08 ± 322.57*	4032.08 ± 30.90*
IL-2	191.94 ± 4.85	907.73 ± 25.97*	706.86 ± 9.94*
IL-4	23.20 ± 0.39	33.25 ± 0.94*	89.82 ± 1.99^∗^•
IL-5	301.70 ± 4.63	879.09 ± 64.19*	365.62 ± 8.61•
IL-6	2.24 ± 0.09	0.65 ± 0.09*	0.52 ± 0.23*
IL-10	56.12 ± 1.67	128.66 ± 2.96*	184.14 ± 7.02^∗^•
IL-17	52.49 ± 2.29	204.85 ± 6.88*	83.41 ± 3.00•
IL-23	66.71 ± 1.13	111.23 ± 3.41*	154.39 ± 3.02^∗^•
TNF-alpha	24.81 ± 0.19	17.80 ± 0.52*	16.51 ± 0.47*
TGF-1beta	6.82 ± 0.12	16.25 ± 0.92*	19.40 ± 0.49*

*p < 0.05 significance of difference compared with the intact control. •p < 0.05 significance of difference compared with the MD.

### Flow Cytometry

**FIGURE 4:**
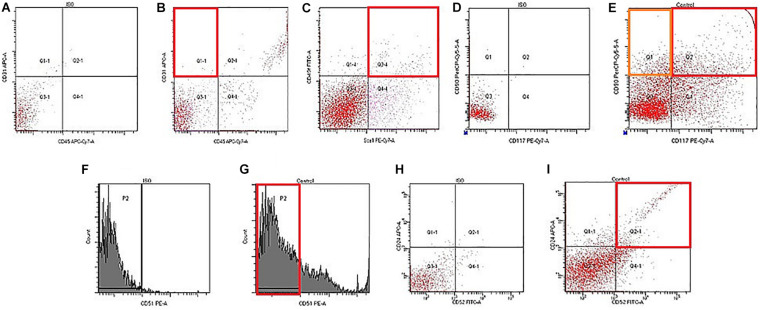
Characterization of endothelial progenitors, epithelial precursors, and spermatogonial stem cells isolated from testis of male C57BL/6 mice on d70. Cells were analyzed by flow cytometry using antibodies against CD45, CD31, Sca1, CD49f, CD117, CD90, CD51, CD24, and CD52. Dot plots are representative for three independent experiments with the mean from three independent experiments. **(A)** Isotype control for IgG2a APC/IgG2b APC-Cy7. **(B)** Phenotype establishment and qualitative analysis of CD31/CD45 expression. **(C)** Phenotype establishment and qualitative analysis of Sca1/CD49f expression. **(D)** Isotype control for IgG2a Per-Cy5.5/IgG2b PE-Cy7. **(E)** Phenotype establishment and qualitative analysis of CD117/CD90 expression. **(F)** Histogram of isotype control for IgG2b PE. **(G)** Histogram of CD51 PE expression. **(H)** Isotype control for IgG2a APC/IgG2b FITC. **(I)** Phenotype establishment and qualitative analysis of CD24/CD52 expression.

**FIGURE 5:**
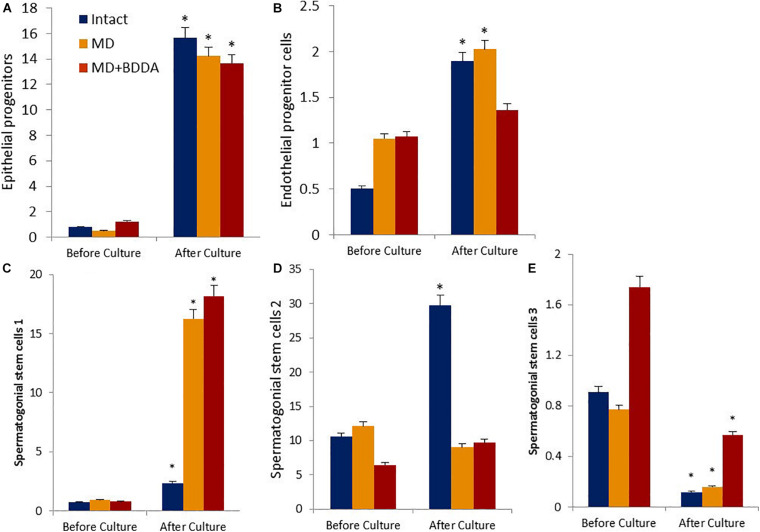
Characterization of epithelial progenitors, endothelial progenitor cells, and spermatogonial stem cells isolated from testes of male C57BL/6 mice on d70 before and after culture (% of all stained mononuclear cells). Cells were analyzed by flow cytometry using antibodies against CD45, CD31, CD34, Sca1, CD49f, CD117, CD29, CD90, CD51, CD24, and CD52. Dot plots are representative for three independent experiments with the mean from three independent experiments. **(A)** The number of epithelial precursors (CD45^–^CD31^–^Sca1^+^CD49f^+^) before and after culture. **(B)** The number of endothelial progenitor cells (CD45^–^CD31^+^ CD34^+^) before and after culture. **(C)** The number of spermatogonial stem cells 1 (CD117^–^CD29^+^CD90^+^) before and after culture. **(D)** The number of spermatogonial stem cells 2 (CD117^+^CD29^+^CD90^+^) before and after culture. **(E)** The number of spermatogonial stem cells 3 (CD51^–^CD24^+^CD52^+^) before and after culture. ^∗^Significance of difference compared with each group before culture (*p* < 0.05). Groups: intact—cells from intact mice, MD—cells from mice with MD, MD + BDDA—cells from mice with MD treated by BDDA.

On d70, flow cytometry was applied to investigate the numbers of stem and progenitor cells in the testes. In group 2, an slightly increased number of pan-hematopoietic cells (CD45^+^) was found, while the population of hemangiogenesis precursors (CD45^–^CD117^+^CD309^+^) and endothelial progenitor cells (EPC) expanded strongly (4.3 times) (Figure 4 and Table 3). Assessment of EPC (CD45^–^CD31^+^CD34^+^) revealed a decrease in their number. According to our data, the number of epithelial precursors with the phenotype (CD45^–^CD31^–^Sca1^+^CD49f^+^) in the testes of group 2 mice decreased, while the number of cells expressing CD326 increased (Figure 5 and Table 3). Moreover, MD reduced the number of SSCs with the phenotype CD117^–^CD29^+^CD90^+^, CD117^+^CD29^+^CD90^+^ in the testes of group 2 compared to group 1. For the number of CD51^–^CD24^+^CD52^+^ SSCs, there was a tendency to decrease (Figure 4 and Table 3).

**TABLE 3:** Content of inflammatory cells, endothelial and epithelial cells, spermatogonial stem cells in the testes of mice with MD on d70 (% of all stained mononuclear cells; M ± m).

Cells	Intact control	Metabolic disorders	Metabolic disorders + BDDA
Pan-hemopoietic cells (CD45^+^)	5.660 ± 0.008	7.416 ± 0.329*	2.839 ± 0.010^∗^•
Precursor cells of hemangiogenesis (CD45^–^CD117^+^Flk1^+^)	0.500 ± 0.007	2.187 ± 0.009*	3.735 ± 0.054^∗^•
Endothelial progenitor cells (CD45^–^CD31^+^CD34^+^)	1.062 ± 0.037	0.541 ± 0.061*	1.468 ± 0.034^∗^•
Epithelial progenitor cells (CD45^–^CD31^–^Sca1^+^CD49f^+^)	0.779 ± 0.281	0.498 ± 0.101*	1.225 ± 0.188^∗^•
Epithelial progenitor cells (CD45^–^CD31^–^CD49f^+^CD326^+^)	0.026 ± 0.011	0.411 ± 0.008*	0.107 ± 0.010^∗^•
Spermatogonial stem cells (CD117^–^CD29^+^CD90^+^)	0.209 ± 0.001	0.133 ± 0.004*	0.140 ± 0.003*
Spermatogonial stem cells (CD117^+^CD29^+^CD90^+^)	7.038 ± 0.003	4.409 ± 0.001*	6.605 ± 0.008^∗^•
Spermatogonial stem cells (CD51^–^CD24^+^CD52^+^)	0.912 ± 0.127	0.767 ± 0.241	1.736 ± 0.240^∗^•

Results of 3 independent experimental series are presented. *p < 0.05 significance of difference compared with the intact control. •p < 0.05 significance of difference compared with MD.

Bisamide Derivative of Dicarboxylic Acid administration significantly reduced the number of pan-hematopoietic cells (by 62%) and epithelial precursors with the phenotype CD45^–^CD31^–^CD49f^+^CD326^+^ (by 74%) in the testes of mice with MD compared to group 2 mice (Figure 4 and Table 3). In contrast, we found an increase in the numbers of hemangiogenesis precursors (by 70%), EPC (by 171%), and CD45^–^CD31^–^Sca1^+^CD49f^+^ epithelial precursors (by 145%) in mice treated with BDDA. Finally, the number of differentiating CD117^+^CD29^+^CD90^+^ SSCs (by 50%) and CD51^–^CD24^+^CD52^+^ SSCs (by 126%) increased, while no significant changes in numbers of self-renewing CD117^–^CD29^+^CD90^+^ SSCs were detected.

### Effects of BDDA on Testicular Stem and Progenitor Cells *in vitro*

Next, we investigated the *in vitro* proliferation of stem and progenitor cells isolated from control mice testes exposed to BDDA and untreated mice with MD. Figure 5 shows that in group 1 the number of CD45^–^CD31^–^Sca1^+^CD49f^+^ epithelial precursors, CD45^–^CD31^+^CD34^+^ EPC, CD117^–^CD29^+^CD90^+^ SSC 1 and CD117^+^CD29^+^CD90^+^ SSC 2 increased significantly by the end of the cultivation. The exceptions were CD51^–^CD24^+^CD52^+^ SSC 3, the number of which decreased by 87%. In group 2, the growth rate of EPC and CD117^+^CD29^+^CD90^+^ SSC 2 was significantly lower than that in the control group (Figure 5 and Supplementary Figure S6). The increase in epithelial precursors, CD117^–^CD29^+^CD90^+^ SSC 1 and CD51^–^CD24^+^CD52^+^ SSC 3 was higher than in group 1. In the group 3 cell culture, we observed a larger increase in cell numbers of all SSCs populations compared to untreated mouse cells with MD (Figure 5). At the same time, proliferation of epithelial precursors and EPC of treated mice with MD was inferior to that in group 2.

## Discussion

The development of MS is associated with obesity, inflammation, and impaired tissue susceptibility to insulin action (insulin resistance). All these factors play a significant role in the development of cardiovascular diseases (arterial hypertension, coronary heart disease) and atherosclerosis. In addition, infertility is known to be one of the complications associated with MS (Freitas Lima et al., 2015; Han and Lean, 2015; Ebrahimi and Christ-Crain, 2016; McCracken et al., 2018).

The pathological effects of obesity on male fertility can be explained by several mechanisms. Firstly, in overweight men, testosterone levels drop due to reduced testosterone synthesis, peripheral conversion of testosterone into estrogens, inhibition of globulin synthesis binding sex steroids, and reduced gonadotropin secretion (Lee et al., 2011). Secondly, in obesity, oxidative stress in the testicles negatively affects spermatogenesis, disrupting the structure of sperm (Wernstedt et al., 2013). Due to the lipid peroxidation in sperm cell membranes, there may be a decrease in sperm motility and a disruption of their interaction with the egg (Kim et al., 2015). Moreover, excess of free radicals could lead to the fragmentation of sperm DNA (Dandona et al., 2005; Simon et al., 2019). In our study, in accordance with the proposed mechanisms listed above, we observed a decrease of free testosterone level, asteno-and oligozoospermia, and infertility (Figure 3).

Several studies reported that MS and its components such as obesity, insulin resistance and dyslipidemia are associated with systemic inflammation which precedes oxidative stress leading to lipid peroxidation (Dandona et al., 2005; Davi and Falco, 2005; Haffner, 2006; Kim et al., 2015) with abnormal changes in the cytokine profiles (Laaksonen et al., 2003; Matsuzawa, 2006; Donath et al., 2010; Wang et al., 2010). In contrast, other studies did neither find changes in blood levels of TNF-α, IL-6, and IL-10 nor an expression of the TNF-α gene in adipose tissue, liver, or skeletal muscle of obese animals (Wernstedt et al., 2013; Kim et al., 2015; Singla et al., 2019). Thus, the cytokine profile may depend on the model of the metabolic syndrome. In the present study, we showed a significant increase in the levels of inflammatory cytokines such as IL-2, IL-4, IL-5, IL-17, and IL-23 in testicular tissue in an MD model (Table 2). Some of these cytokines are produced by M1 macrophages (Gordon et al., 2014; Shi et al., 2018). In this context, we observed high numbers of pan-hematopoietic cells (CD45^+^) in mice with MD (Table 3). This result highlights the potential role of inflammation in the development of astheno- and oligozoospermia and infertility in MD models. Since the serum levels of pro-inflammatory mediators in mice with MD do not exceed the level in intact controls (Skurikhin et al., 2016; Pakhomova et al., 2020), we suggest that MD does not directly lead to systemic inflammation but rather to a local inflammation in testicles of mice with MD. Notably, in the model of MD, the levels of anti-inflammatory IL-4 and IL-10, and TGF-1beta increase simultaneously with the concentration of pro-inflammatory mediators. The cells mainly responsible for secretion of TGF-1beta and IL-10 are alternatively polarized M2 macrophages that have been shown to contribute to resolution of inflammation, wound healing and tissue remodeling via paracrine effects (Borzouei et al., 2019). We postulate that in MD, the ratio between mediators of inflammation and anti-inflammation should be considered. Moreover, assessing both, serum and tissue cytokine concentrations are crucial.

A metabolic syndrome adversely affects all physiological systems including the deleterious effects on the male reproductive system both in metabolic syndrome men and male animals. Male fertility relies on the continuity of spermatogenesis in the testes and spermatogonial stem cells that undergo self-renewal and differentiation compose the “fountainhead” of spermatogenesis (Naifar et al., 2015; Ebrahimi and Christ-Crain, 2016). SSCs are a highly heterogeneous population of testicular stem cells involved in spermatogenesis (Hermo et al., 2010; Zheng et al., 2014). Separation of SSCs is possible based on the expression of CD117, which is a marker of their differentiation (Zhang et al., 2014). Our flow cytometry data indicate a reduction in the numbers of proliferating CD117^–^CD29^+^CD90^+^ SSC and differentiating CD117^+^CD29^+^CD90^+^ SSC in the testicular tissue of mice with MD (Table 3). Interestingly, the number of differentiating CD51^–^CD24^+^CD52^+^ SSC was not significantly affected by MD. In our *in vitro* experiments, CD117^–^CD29^+^CD90^+^ SSC derived from testis of mice with MD generated colonies and their number in culture significantly increased. In contrast, the clonal activity of SSCs (CD51^–^CD24^+^CD52^+^) was lower compared to the intact control where the number of these SSCs in culture was reduced. It is known that the high blood glucose caused by MD changes glucose metabolism in the testis. Leydig cells and SSCs are destroyed under high glucose conditions (Du et al., 2018).

Our data indicate high functional activity of SSCs with the phenotypes CD117^–^CD29^+^CD90^+^ and CD117^+^CD29^+^CD90^+^ as well as their potential participation in spermatogenesis in MD. Under normal glycose conditions and with a lipid profile of healthy animals, the activity of CD117^+^CD29^+^ CD90^+^ SSC and CD117^–^CD29^+^CD90^+^ SSC is sufficient to maintain spermatogenesis.

Destructive changes in the tubular apparatus of the testes and their microvascular network in mice with MD were the factors initiating the regeneration of endothelium and epithelium. This is evidenced by an increase in tissue-specific epithelial precursors (CD45^–^CD31^–^CD49f^+^CD326^+^) (Table 3). We attribute the reduction in the population of CD45^–^CD31^+^CD34^+^ EPC and CD45^–^CD31^–^Sca1^+^CD49f^+^ epithelial precursors in the testes of mice of group 2 to inflammation, obesity, and diabetes. Meanwhile, even the small number of surviving CD45^–^CD31^+^CD34^+^ EPC and CD45^–^CD31^–^Sca1^+^CD49f^+^ epithelial precursors under MD conditions show a high activity *in vitro* and/or *in vivo* (Table 3).

In our experiments, the application of BDDA caused an increase in the serum testosterone concentration, increased the number of layers of spermatogenic epithelium in the seminiferous tubules and the number of mobile forms of sperm, and positively influenced fertility of animals with MD (Figure 3).

This positive impact of BDDA treatment of hypogonadism in male C57BL/6 mice with MD was unexpected. We hypothesized that the effects of BDDA are mediated by tissue-specific stem cells. Our flow cytometric analysis revealed an increase of EPCs (CD45^–^CD31^+^CD34^+^), hemangiogenic precursors (CD45^–^CD117^+^CD309^+^), epithelial precursors (CD45^–^CD31^–^Sca1^+^CD49f^+^), and differentiating SSCs in the testes of mice with MD treated with BDDA compared to group 2 (Figure 5 and Table 3). SSCs of group 3 exhibited higher clonal activity *in vitro* compared to group 2. Thus, a possible mechanism of action of BDDA could be a stimulation of SSC and EPC. This could also be explained by the anti-inflammatory activity of BDDA. This hypothesis is supported by a decrease in the level of pro-inflammatory cytokines in the testicular tissue during the treatment with BDDA (Table 2). In this context, BDDA could induce a shift in the activity of the immune system toward an increase in the production of anti-inflammatory cytokines.

Despite the positive effects of BDDA, we understand that our metabolic disorder model does not fully reproduce the clinical picture of the diseases (metabolic syndrome and diabetes). The clinical picture of the disease is much more complicated. This inhibits immediate clinical use of BDDA. Therefore, the study of BDDA is needed in other experimental models of metabolic syndrome and diabetes.

## Conclusion

Our study provides evidence that BDDA improves the physiological functions of SSCs and thus enhances the regeneration of germ cells and restores the fertility of animals with MD. The positive effect of the treatment can be partially explained by a decrease in dyslipidemia and obesity, a break in the causal relationship between obesity and inflammation, as well as between obesity, insulin resistance, and diabetes mellitus. Our results suggest that BDDA could be a promising therapeutic agent to restore the functions of the reproductive system in male patients with MD.

## Data Availability Statement

All datasets presented in this study are included in the article/Supplementary Material.

## Ethics Statement

The animal study was reviewed and approved by the Laboratory Animal Control Committee of the Goldberg ED Research Institute of Pharmacology and Regenerative Medicine, Tomsk NRMC (IACUC Protocol No. 114062016).

## Author Contributions

ES: conceptualization, writing—original draft preparation, supervision, project administration, and funding acquisition. OP, AP, and ES: methodology and data curation. EP: software. OP, AP, and NE: validation. OP and ES: formal analysis. AP, OP, VK, LS, EP, and NE: investigation. VN and AD: resources. OP, DW, and ES: writing—review and editing. VK, LS, and EP: visualization. All authors have read and agreed to the published version of the manuscript.

## Conflict of Interest

VN was employed by the company “PHARMENTERPRISES” (Moscow, Russia). The remaining authors declare that the research was conducted in the absence of any commercial or financial relationships that could be construed as a potential conflict of interest.

## Abbreviations

BDDA: bisamide derivative of dicarboxylic acid
BMI: body mass index
DMEM: Dulbecco’s modified Eagle’s medium
EPC: endothelial progenitor cells
HDL: high-density lipoproteins
IL: interleukin
MS: metabolic syndrome
LDL: low-density lipoproteins
MD: metabolic disorders
PBS: phosphate buffered saline
SSC: spermatogonial stem cells
SC: stem cells
TG: triglycerides
TGF-1β: transforming growth factor beta
TNF-α: tumor necrosis factor alpha
VLDL: very low-density lipoproteins.
