# Supplementary material for: Bisamide Derivative of Dicarboxylic Acid Contributes to Restoration of Testicular Tissue Function and Influences Spermatogonial Stem Cells in Metabolic Disorders
Source: Front Cell Dev Biol. 2020 Dec 3;8:562358. doi: 10.3389/fcell.2020.562358 (PMC7744787; doi:10.3389/fcell.2020.562358)
Supplement: Supplementary file 1 [file Table_1.DOCX]

Supplementary Material

# Supplementary Tables

**Table 1S**. Summary of food consumption by male С57ВL/6 mice during 70-days period of the experiment in day (g/mice, M±m).

| **Group** | **d28** | **d49** | **d70** |
| --- | --- | --- | --- |
| Intact control | 2.15±0.19 | 2.27±0.16 | 2.38±0.24 |
| Metabolic Disorders | 2.23±0.24 | 3.04±0.21* | 3.41±0.24* |
| Metabolic Disorders + BDDA |  |  | 2.75±0.38 |

* - p<0.05 significance of difference compared with the intact control.

# Supplementary Figures


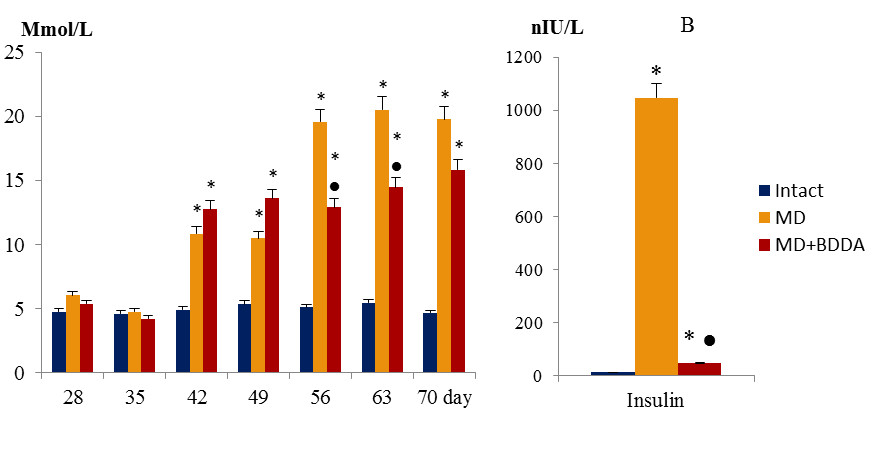


**Supplementary Figure 1.** The effect of BDDA on the parameters of the metabolic syndrome of male C57BL/6 mice. (**A**) The glucose level in serum of mice within 70 days of the experiment (Mmol/l); (**B**) insulin level in serum of mice on d70 (nIU/L). Groups: intact – a control group from intact mice, MD – mice with MD, MD+BDDA – mice with MD treated BDDA. Results are presented as the mean ± SEM. * - significance of difference compared with intact (p < 0.05); ● - significance of difference compared with the MD group (p < 0.05).


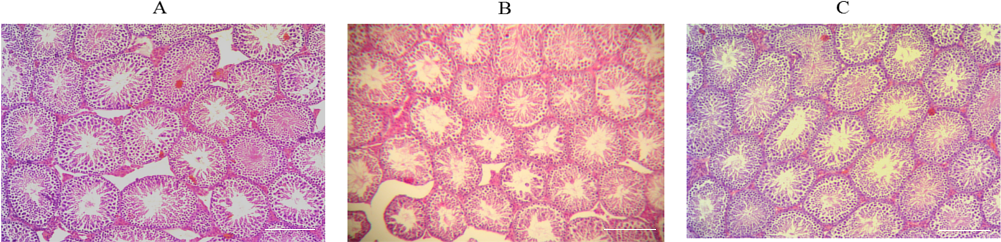


**Supplementary Figure 2.** Photomicrographs of representative testis sections obtained from male C57BL/6 mice on d70. Tissue was stained with haematoxylin-eosin. (**A**) Section from control group; (**B**) Section from mice with MD; (**C**) Section from mice with MD treated with BDDA between d49-d70. At least 10 photomicrographs of the testicular tissue at × 100 magnification were taken for each experimental animal from all experimental groups (group 1, 2 and 3).


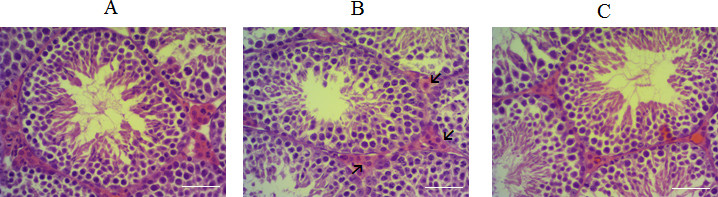


**Supplementary Figure 3.** Photomicrographs of representative testis sections obtained from male C57BL/6 mice on d70. Tissue was stained with haematoxylin-eosin. (**A**) Section from control group; (**B**) Section from mice with MD; (**C**) Section from mice with MD treated with BDDA between d49-d70. At least 10 photomicrographs of the testicular tissue at × 400 magnification were taken for each experimental animal from all experimental groups (group 1, 2 and 3). Scale bar 50μm. The arrows indicate cytoplasm vacuolization of Leydig cells.


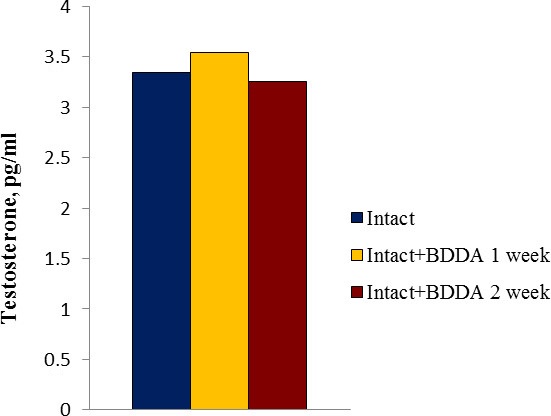


**Supplementary Figure 4.** The free testosterone level in the serum of mice. Groups: Intact – healthy male mice; Intact + BDDA 1 week - healthy male mice after BDDA administration during 1^st^ week; Intact + BDDA 2 week - healthy male mice after BDDA administration during 2^nd^ weeks.


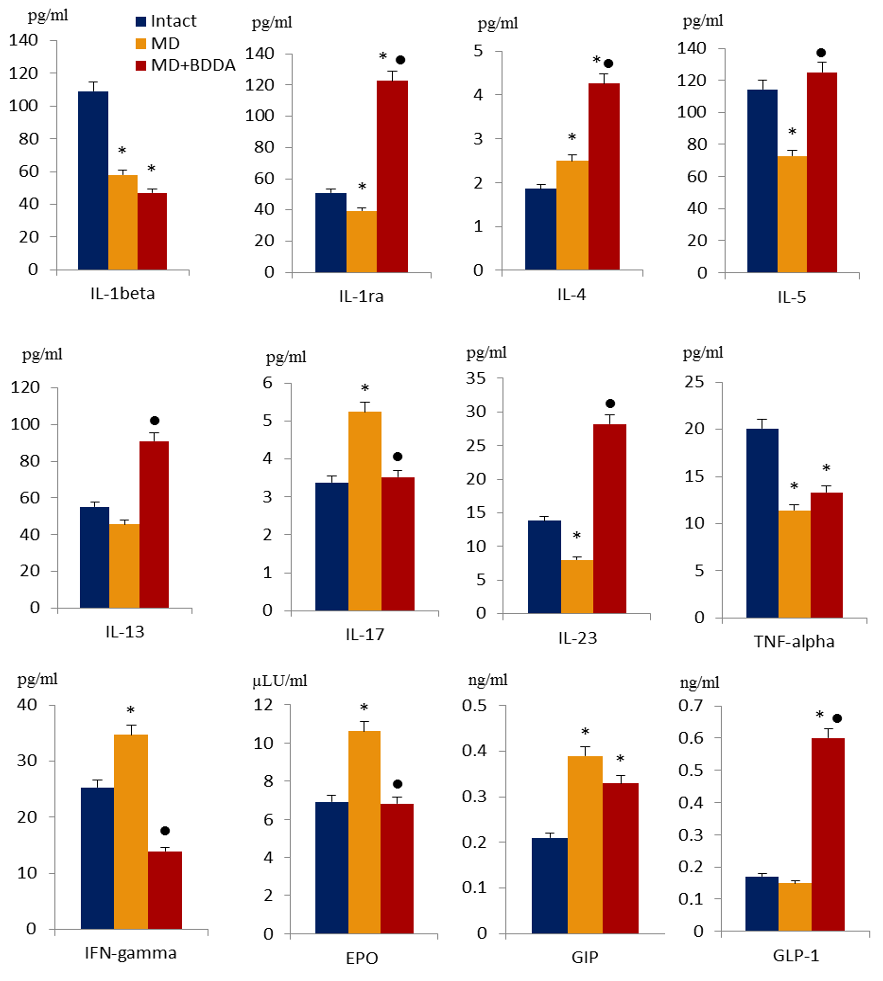


**Supplementary Figure 5.** The level of interleukins (1beta, 4, 5, 13, 17, 23), IL-1ra, TNF-alpha, IFN-gamma, EPO, GIP and GLP-1 in the serum of male C57BL/6 mice at d70. Groups: intact—a control group from intact mice, MD—mice with metabolic disorders (MD), MD + BDDA—mice with MD treated BDDA. Results are presented as the mean ± SEM. *: significance of difference compared with intact (p<0.05); ●: significance of difference compared with the MD group (p < 0.05).


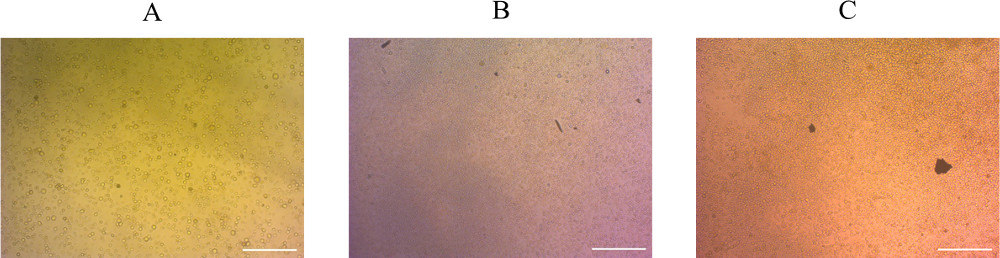


**Supplementary Figure 6.** Open field photographs of the cell culture isolated from testis of male C57BL/6 mice on d70 after 7 days of culture. (**A**) Cell culture from the control group; (**B**) Cell culture from mice with MD; (**C**) Cell culture from mice with MD treated with BDDA between d49-d70. Scale bar 200 μm.
